# LocalNgsRelate: a software tool for inferring IBD sharing along the genome between pairs of individuals from low-depth NGS data

**DOI:** 10.1093/bioinformatics/btab732

**Published:** 2021-10-28

**Authors:** Alissa L Severson, Thorfinn Sand Korneliussen, Ida Moltke

**Affiliations:** Department of Genetics, Stanford University, Stanford, CA 94305-5020, USA; GLOBE Institute, University of Copenhagen, 1350 Copenhagen K, Denmark; Department of Biology, University of Copenhagen, 2200 Copenhagen N, Denmark

## Abstract

**Motivation:**

Inference of identity-by-descent (IBD) sharing along the genome between pairs of individuals has important uses. But all existing inference methods are based on genotypes, which is not ideal for low-depth Next Generation Sequencing (NGS) data from which genotypes can only be called with high uncertainty.

**Results:**

We present a new probabilistic software tool, LocalNgsRelate, for inferring IBD sharing along the genome between pairs of individuals from low-depth NGS data. Its inference is based on genotype likelihoods instead of genotypes, and thereby it takes the uncertainty of the genotype calling into account. Using real data from the 1000 Genomes project, we show that LocalNgsRelate provides more accurate IBD inference for low-depth NGS data than two state-of-the-art genotype-based methods, Albrechtsen *et al.* (2009) and hap-IBD. We also show that the method works well for NGS data down to a depth of 2×.

**Availability and implementation:**

LocalNgsRelate is freely available at https://github.com/idamoltke/LocalNgsRelate.

**Supplementary information:**

Supplementary data are available at *Bioinformatics* online.

## 1 Introduction

Inference of identity-by-descent (IBD), i.e. allelic identity due to recent common ancestry, along the genome for pairs of individuals has proven useful in several research fields, ranging from medical genetics, where it can be used to map disease associated genes (Albrechtsen *et al.*, 2009) to evolutionary genetics where it can be used to detect natural selection (Albrechtsen *et al.,* 2010). There are numerous methods for such inference, e.g. the Hidden Markov Model (HMM)-based method by Albrechtsen *et al.* (2009) and hap-IBD (Zhou *et al.*, 2020). However, they are all based on genotype data, which makes them ill-suited for the increasingly common low-depth Next Generation Sequencing (NGS) data, because genotypes cannot be called with high certainty for such data (O’Rawe *et al.*, 2015). Motivated by this, we here present a new HMM-based method, LocalNgsRelate. It is similar to Albrechtsen *et al.* (2009), but instead of basing its inference on genotypes and taking genotype uncertainty into account via a global user-supplied error rate, it bases its inference on genotype likelihoods (GLs) and accounts for genotype uncertainty via these, like NgsRelate (Korneliussen and Moltke, 2015). To investigate its performance, we apply it to low-depth NGS data from the 1000 Genomes project and compare it to Albrechtsen *et al.* (2009) and hap-IBD.

## 2 Materials and methods

At any given genomic locus in a diploid species, two noninbred individuals *i* and *j* share either 0, 1 or 2 alleles IBD. Based on NGS data, we aim to be able to infer this IBD state at each locus along the genome using the following framework: let $D^{i}=(D_{1}^{i},...,D_{L}^{i})$ and $D^{j}=(D_{1}^{j},...,D_{L}^{j})$ denote the observed NGS data for *i* and *j* at *L* diallelic loci and $G^{i}=(G_{1}^{i},...,G_{L}^{i})$ and $G^{j}=(G_{1}^{j},...,G_{L}^{j})$ denote their true unobserved genotypes at these same loci. Also, let *A* and *a* denote the two alleles at locus *l* and $f^{A}=(f_{1}^{A},...,f_{L}^{A})$ denote the frequencies of *A*. Finally, let $X=(X_{1},...,X_{L})$ denote the unobserved IBD states each with the value 0, 1 or 2. As in Albrechtsen *et al.* (2009), we then assume *X* can be described using a continuous time Markov chain with instantaneous rate matrix

$$Q=\left(\begin{array}{ccc} -\alpha k_{1} & \alpha k_{1} & 0\\ \alpha k_{0} & -\alpha (k_{0}+k_{2}) & \alpha k_{2}\\ 0 & \alpha k_{1} & -\alpha k_{1} \end{array}\right),$$

where $R=(k_{0},k_{1},k_{2})$ are the relatedness coefficients, i.e. the genome-wide proportion of loci where the two individuals share 0, 1 and 2 alleles IBD, respectively, and where *α* is the overall rate of change in IBD state. This model has *R* as stationary distribution and leads to closed form expressions for transition probabilities that depend on the distance between consecutive loci, $P(X_{l+1}|X_{l},R,\alpha )$ (Albrechtsen *et al.*, 2009). We also assume that the genotypes are independent conditional on the IBD states, i.e. that there is no linkage disequilibrium (LD), and that the frequencies *f^A^* are known. Under these assumptions, the likelihood $P(D^{i},D^{j}|R,\alpha )$ can be written as the following sum over all possible values *x* of *X*
 

$$\sum_{x}(P\left(X_{1}=x_{1}|R\right)\prod_{l=2}^{L}P\left(X_{l}=x_{l}|X_{l-1}=x_{l-1},R,\alpha \right)\times \prod_{l=1}^{L}\sum_{\left(G_{l}^{i},G_{l}^{j}\right)}P(G_{l}^{i}|f_{l}^{A})P(G_{l}^{j}|f_{l}^{A},X_{l}=x_{l},G_{l}^{i})P(D_{l}^{i}|G_{l}^{i})P(D_{l}^{j}|G_{l}^{j})),$$

where $P(G_{l}^{i}|f_{l}^{A})$ and $P(G_{l}^{j}|f_{l}^{A},X_{l}=x_{l},G_{l}^{i})$ are given in Supplementary Tables S1 and S2 and $P(D_{l}^{i}|G_{l}^{i})$ and $P(D_{l}^{j}|G_{l}^{j})$ are GLs. Based on this, we first perform maximum likelihood estimation of *R* and *α* with the numerical optimization algorithm BFGS and then make inferences about the IBD states, *X*, with standard HMM algorithms Viterbi and posterior decoding. For details about the performance assessment, including example run times, see Supplementary Data.

## 3 Results

**Fig. 1.**
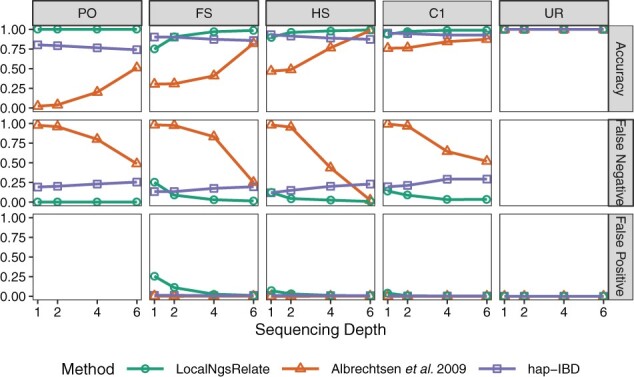
Performance of LocalNgsRelate, Albrechtsen *et al.* (2009) and hap-IBD for five pairs of individuals at different sequencing depths. Performance was assessed by comparing the IBD state assigned by each method when applied to NGS data to the IBD state assigned by Albrechtsen *et al.* (2009) applied to high-quality genotype data for the same individuals

We investigated the performance of LocalNgsRelate on 1000 Genomes NGS data for five pairs of LWK individuals with different degrees of relatedness: a parent-offspring (PO), a full-sibling (FS), a half-sibling (HS), a first cousin (C1), and an unrelated (UR) pair and used NGS data from 94 additional LWK individuals for allele frequency estimation (Supplementary Table S3). We downsampled the NGS data from ∼6× to 4×, 2× and 1×, estimated GLs and allele frequencies at each sequencing depth using ANGSD (Korneliussen *et al.*, 2014), and applied LocalNgsRelate. Then we compared these results to those from Albrechtsen *et al.* (2009), applied to high-quality genotype data from the same samples, which we used as a proxy for the truth. We performed this comparison using a set of loci obtained by quality filtering and LD pruning the high-quality genotype data. Encouragingly, LocalNgsRelate accurately estimates *R* (Supplementary Fig. S1) as well as IBD states down to depths of at least 2× (Fig.  1). Moreover, performing the same assessment of Albrechtsen *et al.* (2009) and hap-IBD applied to genotypes called from the low-depth NGS datasets showed that LocalNgsRelate has higher accuracy, lower false negative rates, and only modestly higher false positive rate than both these methods for all related pairs (Fig.  1 and Supplementary Fig. S3A). As Albrechtsen *et al.* (2009) takes a user-supplied genotype error rate, we not only performed this comparison using the default error rate, but also a range of other values. As expected, the performance of Albrechtsen *et al.* (2009) depends on the error rate; however, the optimal error rate depends both on sequencing depth and the degree of relatedness (Supplementary Figs S4 and S5) and in all cases LocalNgsRelate performed better.

Next, we considered the effect of reduced sample size, which impacts allele frequency estimation, on LocalNgsRelate. From the 101 NGS samples, we took subsets of size 50, 25, 15 and 7 (including the 7 samples that make up the related pairs), and repeated the analyses (Supplementary Fig. S6). Interestingly, the performance is not affected much until only 15 samples are available. Then the accuracy starts to decrease, especially at 1× depth. Similarly, we investigated how robust the method is to the use of allele frequencies from other populations more or less similar to the population the samples of interest are from, here represented by YRI and CEU, respectively. Not surprisingly, this showed that the method can be used with allele frequencies from a similar population without much loss in performance, but that its performance is markedly decreased if the allele frequencies are from a less similar population (Supplementary Fig. S7).

We also evaluated the importance of accurate LD pruning for the performance of LocalNgsRelate. To do so we called SNPs from the NGS data, and then thinned the new SNPs based on distance as an approximation for LD pruning. When we applied LocalNgsRelate to this set of SNPs instead we did not see a substantial performance effect, and this result was robust across several choices of distances (Supplementary Figs S8–S11). If anything, at lower depths and samples sizes LocalNgsRelate actually performs better on this less accurately LD pruned dataset.

Finally, we note that the above results for LocalNgsRelate were obtained using Viterbi inference; however, when using posterior decoding and a posterior cutoff, we observed comparable results (Supplementary Figs S12 and S13).

## 4 Discussion and conclusion

We have presented LocalNgsRelate, a new method for inferring IBD sharing along the genome of two individuals. We have shown that it performs well and outperforms the state-of-the-art genotype-based methods Albrechtsen *et al.* (2009) and hap-IBD on low-depth NGS data. For hap-IBD, this result is not surprising as this method is designed for phased genotype data, and, like genotype calling, phasing is difficult for low-depth NGS data. In fact, hap-IBD’s performance is likely markedly worse when, unlike in our example, there is no reference panel available for phasing, as is often the case for nonmodel organisms. On the other hand, to be fair it should be noted that the hap-IBD results are based on genotypes imputed using GLIMPSE and thus part of the reduced performance may be due to the performance of GLIMPSE rather than hap-IBD itself.

For Albrechtsen *et al.* (2009), the result is perhaps more surprising since this method basically only differs from LocalNgsRelate in the way genotype uncertainty is handled. However, our results suggest that using GLs not only makes a user-supplied error rate obsolete, it also better reflects the nature of uncertainty in low-depth NGS data. It should be noted that Albrechtsen *et al.* (2009) has one feature that LocalNgsRelate does not: an ad hoc correction for LD based on LD estimates obtained from genotype data. LocalNgsRelate instead requires LD pruned data. Because it is challenging to estimate LD from low-depth NGS data (Fox *et al.*, 2019), it is difficult to use the same correction as Albrechtsen *et al.* (2009) and it may even be difficult to prune for LD. However, encouragingly, we obtained comparable results when we LD pruned simply by thinning SNPs based on distance, suggesting that LocalNgsRelate can be used even when LD cannot be estimated.

## Funding

A.L.S. was supported by the NSF Graduate Research Fellowship and the NIH [R01 HG005855]. I.M. was funded by the European Research Council [ERC-2018-STG-804679]. 

*Conflict of Interest*: none declared. 

## Supplementary Material

btab732_supplementary_data
