# Supplementary material for: LocalNgsRelate: a software tool for inferring IBD sharing along the genome between pairs of individuals from low-depth NGS data
Source: Bioinformatics. 2021 Oct 28;38(4):1159–61. doi: 10.1093/bioinformatics/btab732 (PMC8796377; doi:10.1093/bioinformatics/btab732)
Supplement: btab732_supplementary_data [file btab732_supplementary_data.pdf]

Supplementary Data

Alissa L. Severson, Thorfinn S. Korneliussen and Ida Moltke

October 27, 2021

Contents

1 Supplementary Text **2**
1.1 A detailed description of the performance test datasets 2
1.1.1 Samples 2
1.1.2 Genomic data 2
1.2 A detailed description of the performance assessment analyses 2
1.2.1 Application of Albrechtsen *et al.* (2009) to HQ genotype data to obtain a proxy for the true IBD states 2
1.2.2 Application of LocalNgsRelate to low-depth NGS data 3
1.2.3 Application of LocalNgsRelate to low-depth NGS data (distance thinned SNP set) . . 4
1.2.4 Application of Albrechtsen *et al.* (2009) to genotypes called from low-depth NGS data (using ANGSD) 5
1.2.5 Application of Albrechtsen *et al.* (2009) to genotypes called from low-depth NGS data (using GATK) 6
1.2.6 Application of hap-IBD to genotypes called from low-depth NGS data 6
1.2.7 Using allele frequency estimates from other populations 7

2 Supplementary Tables **9**

3 Supplementary Figures **10**

References **23**

1 Supplementary Text

1.1 A detailed description of the performance test datasets

1.1.1 Samples

To assess the performance of LocalNgsRelate we used data from the 1000 Genomes, specifically individuals from the LWK population, since this population sample is known to contain several close relatives. We focused our analyses on 5 pairs of individuals with different degrees of relatedness (Table S3) made up of a total of 7 individuals, but also used up to 94 other LWK individuals for allele frequency estimation and LD estimation.

1.1.2 Genomic data

As part of the 1000 Genomes project the LWK individuals were both whole genome sequenced at low-depth and densely genotyped on the Illumina Omni chip. We used both data types.

Array based genotype data
We downloaded a vcf file containing the high quality genotypes from the ftp server at

`ftp://ftp.1000genomes.ebi.ac.uk/vol1/ftp/release/20130502/supporting/hd_genotype_chip/`

The dataset included 116 individuals, including the 101 whole genome sequenced individuals. With plink v1.9 [3], we filtered for di-allelic variants with a minor allele frequency greater than 0.05, resulting in 1,413,513 variants. Next we pruned for LD, using `--indep-pairwise` in plink with a window size of 50 variants, setting the step size to 10 variants, and a pairwise r^2 threshold of 0.1. This pruning resulted in 125,058 SNPs which were used for all analyses of the array data. We will refer to this dataset as the high quality (HQ) genotype data below.

Whole genome data
We downloaded all 101 low-depth sequenced whole genomes that were available for individuals from the LWK population on the ftp server at

`ftp://ftp.1000genomes.ebi.ac.uk/vol1/ftp/phase3/data/`.

The 1000 Genomes sequenced samples to a minimum depth of 3X, and the samples have an average depth of 6.2X (Table S3).

1.2 A detailed description of the performance assessment analyses

1.2.1 Application of Albrechtsen *et al.* (2009) to HQ genotype data to obtain a proxy for the true IBD states

Input data
To be able to assess the performance of LocalNgsRelate we needed the true IBD states for our pairs of samples. Since these are not available, we applied Albrechtsen *et al.* (2009) to the quality filtered and LD pruned genotype dataset described above and used the inferred IBD states as proxies for the true states.

Inference using Albrechtsen *et al.* (2009)
Because the input data were already LD pruned, we never used the ad hoc LD correction available in Albrechtsen *et al.* (2009) (i.e. we set `back=0, ld_adj=FALSE`). In addition, for all pairs except for full siblings (FS) we set $k_2 = 0$ with `fix.k2=0`.

We initially explored four different program options in Albrechtsen *et al.* (2009) to determine the optimal settings for estimation of $R = (k_0, k_1, k_2)$ and α. First, we used the default settings which calculate the rate parameter α. Next we changed the method to estimate α (`calc.a=FALSE`), and used the default limits (`alim=c(0.001, 0.15)`). Then we lowered the upper limit of α to 0.08 (`alim=c(0.001, 0.08)`).

Finally to test the effect of the error rate ϵ on the results we doubled it from the default value 0.01 to 0.02 (`epsilon=0.02`).

After the optimal program options were chosen, we compared several possible IBD state inference approaches. Albrechtsen *et al.* (2009), like LocalNgsRelate, provides two algorithms for inferring IBD states along the genome: the Viterbi algorithm, which provides the most likely path of IBD states along the genome, and posterior decoding that provides a posterior probability for each of the three possible IBD states at each locus. These three posterior probabilities can subsequently be used to assign IBD state, e.g. by assigning the IBD state with a posterior probability above some cutoff value or 'no call' if none of the states have a posterior above the cutoff. We considered the Viterbi algorithm and posterior decoding with two different posterior probability cutoffs, a more lenient value of 0.75 and a more stringent cutoff of 0.95.

Results

The results of exploring the different program options are shown in Table S4. Comparing the effect of these on the estimates of the parameters $R = (k_0, k_1, k_2)$ and α, we found that limiting α and increasing ϵ did not markedly change the estimates for the pairs. We therefore chose to use default values for these. Hence the only deviation from default for the four explored program options we decided to make in our analyses was to estimate α instead of calculating it as this gave a better fit to the expected k_0, k_1 and k_2 values.

When using these chosen program options the comparison of the IBD states inferred by Viterbi and posterior decoding with two different posterior cutoffs for each related pair, showed high concordance. Specifically, when treating the results from the Viterbi algorithm as the true IBD states, both the false positive and false negative rates are low for the results based on posterior decoding (Fig. S14). In general, we saw that the discordant calls between Viterbi and the posterior decoding based approach were mainly due to loci, which did not meet the posterior cutoff and were thus assigned as 'no call' (i.e. when neither of the IBD states 0, 1 or 2 has a posterior probability above the cutoff and thus no IBD state is assigned). As expected the false positive and false negative rates are slightly higher when using a posterior cutoff of 0.75 versus 0.95, however this came at the cost of more loci being assigned as 'no call'. Because the concordance rate was so high between Viterbi and the posterior cutoffs, and because Viterbi assigns an IBD state at each SNP without needing a user supplied cutoff, we choose to use the IBD states inferred by applying Viterbi to the HQ genotype data as a proxy for the true states in all later analyses.

1.2.2 Application of LocalNgsRelate to low-depth NGS data

Input data

To assess the effect of sequencing depth and sample size on the performance of LocalNgsRelate we created input data, including GLs and allele frequencies, in a stepwise manner. First, we downsampled the bam files to 4X, 2X, and 1X using `samtools view` with the flag `-s` [2]. Then, for each sequencing depth we used ANGSD [1] to estimate genotype likelihoods and allele frequencies at the same 125,058 SNPs as in the HQ genotype data, which are not in LD. For each sequencing depth, and for sample sizes of $n = 7, 15, 25, 50, 101$ individuals, we estimated minor allele frequencies and genotype likelihoods with the options `-GL 1`, `-doMajorMinor 3`, `-doMaf 1`, `-doGlf 2`, `-minMapQ 30`, `-minQ 20`. Note that all datasets included the 7 individuals in the related pairs that the IBD inference was applied to and $n - 7$ randomly sampled additional LWK samples.

Inference using LocalNgsRelate

We applied LocalNgsRelate to each of the 20 combinations of sample size and sequencing depth. We ran LocalNgsRelate with default settings, except that for all pairs except FS we fixed $k_2 = 0$. For the IBD state inference we primarily used the Viterbi algorithm, but we also performed an analysis using posterior decoding combined with a posterior probability cutoff of 0.75.

Assessing inference output

To assess the inference results from LocalNgsRelate we compared them to the results from Albrechtsen *et al.* (2009) applied to the HQ genotype data. Because our genotype likelihoods were estimated at the same SNP loci as the HQ genotype data, for all loci we could directly compare the IBD states called by the

Viterbi algorithm in LocalNgsRelate to the IBD states called by Albrechtsen *et al.* (2009). We evaluated the accuracy, false postive rate, and false negative rate of LocalNgsRelate by taking the IBD state assigned by the method as the predicted state, and comparing it to the IBD state assigned by Albrechtsen *et al.* (2009), which we assume to be the true state. Under this scheme, the accuracy is the fraction of loci where the IBD state assigned by LocalNgsRelate matches the assignment by Albrechtsen *et al.* (2009). The false negative rate is the fraction of loci where LocalNgsRelate assigned a lower IBD state than Albrechtsen *et al.* (2009). And finally the false positive rate is the fraction of loci where LocalNgsRelate assigned a higher IBD state than Albrechtsen *et al.* (2009).

We also evaluated the performance of LocalNgsRelate with posterior decoding, using a posterior probability cutoff of 0.75 (Fig. S12). Note that in this case, if the posterior probability at a locus does not exceed the cutoff for any of the 3 possible IBD states, then the locus was designated a 'no call'. We used a 0.75 cutoff to minimize the 'no call' rate.

Results

The results of the Viterbi based performance assessment are shown in Figs. S1 and S6. The results of the performance of LocalNgsRelate when instead using posterior decoding combined with a posterior probability cutoff of 0.75 is shown in Fig. S12. Each run of LocalNgsRelate was given 2 Gb of memory, and runtimes of LocalNgsRelate are seen in Figure S15. The majority of runtimes are less than a minute, and all are less than two minutes.

1.2.3 Application of LocalNgsRelate to low-depth NGS data (distance thinned SNP set)

Input data

To investigate how much the performance of LocalNgsRelate is affected by not accurately pruning for LD, we also made another set of input data by applying the following stepwise procedure to each of the 20 different combinations of depths and samples sizes from the original NGS based analysis: First we called SNPs directly from the low-depth bam files, and estimated allele frequencies and temporarily called genotypes for these SNPs with ANGSD [1] using the flags: `-GL 1`, `-doMajorMinor 1`, `-doMaf 2`, `-doPlink 2`, `-doGeno -4`, `-doPost2`, `-doCounts 1`, `-doGlf 2`, `-minMapQ 30`, `-minQ 20`, `-uniqueOnly 1`, `-remove bads 1`, `only proper pairs 1`, and selecting SNPs with filters `-SNP-pval 1e-6`, `-minMAF 0.05`, and `-minInd 3`.

Next, in a simple attempt to prune for LD without estimating LD, we thinned the obtained set of SNPs based on distance with plink [3]. Specifically, we chose to match the number of variants in the previous sections, approximately 125,000, and removed SNPs using the options `--geno 0.3`, to filter for missingness greater than 30%, and `--bp-space 20000` to thin the SNPs to 1 per 20 Kb, leaving approximately 125,000 SNPs. We note that because less SNPs are called at 1X depth, the number of SNPs is lowest in those cases. To assess the effect of thinning to different SNP densities, we also created a dataset with more SNPs by thinning to 1 per 5 and 10 Kb, leaving approximately 250,000 and 500,000 SNPs respectively. With our three sets of thinned SNPs, we subsetted the genotype likelihoods and allele frequencies already obtained with ANGSD command described above and used them as input for LocalNgsRelate.

Inference using LocalNgsRelate

We applied LocalNgsRelate to each related pair, at each sequencing depth, and for each sample size. We did so using the same settings as when we applied LocalNgsRelate to the properly LD pruned datasets: for all pairs except for the full sibling pair we fixed $k_2 = 0$, and otherwise we used the default settings. To infer IBD states along the genome, we primarily used the Viterbi algorithm. In addition, we also tried to use posterior decoding combined with a posterior cutoff of 0.75.

Assessing inference output

As in section 1.2.2, we assessed the results by comparing them to the results from Albrechtsen *et al.* (2009) applied to the HQ genotype data. However, unlike in section 1.2.2, the SNP loci used in this analysis do not exactly correspond to those in the HQ genotype data. To compare the results for a SNP in this analysis that was not in the HQ dataset, we first determined if it is located between two SNPs in the HQ genotype dataset that are inferred to have different IBD states using Albrechtsen *et al.* (2009). If this is the case, then the

SNP is categorised as a 'mismatch', because we cannot confidently assign the SNP a true IBD state. If this is not the case, then we compared the inferred IBD state of this SNP to the IBD state of the nearest SNP to both sides in the HQ dataset inferred by Albrechtsen *et al.* (2009). This procedure allowed us to calculate accuracy, false positive rate, and false negative rate as before (section 1.2.2), with the only difference being that in this case we also have a mismatch rate.

Results
The estimated $R = (k_0, k_1, k_2)$ for each related pair are shown in Fig. S8, and the result of comparing LocalNgsRelate's Viterbi based IBD state inference to that of Albrechtsen *et al.* (2009) applied to the HQ genotype data can be found in Fig. S9.

Fig. S10-S11 shows the performance of LocalNgsRelate when applied to a larger dataset, thinned to 250,000 and 500,000 SNPs, respectively. Despite doubling/quadrupling the number of SNPs, we see little change in the performance of the method. However, we do observe that the increased number of SNPs, which are more likely to be in LD with each other, does slightly increase the rate of false positives for higher sequencing depths.

In Fig. S13 we show the performance of LocalNgsAlbrechtsen *et al.* (2009) when calling IBD state with a posterior cutoff of 0.75 from the dataset thinned to approximately 125,000 SNPs.

1.2.4 Application of Albrechtsen *et al.* (2009) to genotypes called from low-depth NGS data (using ANGSD)

Input data
To investigate the performance of Albrechtsen *et al.* (2009) compared to LocalNgsRelate when only low depth NGS data is available we applied Albrechtsen *et al.* (2009) to genotypes called from NGS data of each of the four sequencing depths at the 125,058 SNPs in the HQ genotype data. Specifically, we first called genotypes with ANGSD [1] and output the data in .tped, using options `-doPlink 2`, `-doGeno -4`, `-doPost 2`, `-doMajorMinor 3`, `-GL 1`, `-doCounts 1`, `-doMaf 1`, `-minMapQ 30`, `-minQ 20`. Next, because Albrechtsen *et al.* (2009) estimates allele frequencies from the provided genotypes, we merged the called genotypes of the 7 related individuals with the HQ genotypes for the remaining 109 LWK individuals. In doing this we minimize the impact of allele frequency estimation on Albrechtsen *et al.* (2009)'s performance, isolating the effect of error in genotypes called from low-depth NGS data. However, we note that the results presented here therefore do not reflect any potential errors that could be caused by not having high quality genotypes to estimate allele frequencies from.

Inference using Albrechtsen *et al.* (2009)
For each of the four sequencing depths, we applied Albrechtsen *et al.* (2009) to the called genotypes with 6 different error rates: $\epsilon = 0.01, 0.02, 0.05, 0.1, 0.2, 0.5$. An error rate of 0.01 is the default, but we tried the other error rates as well because Albrechtsen *et al.* (2009) allows the user to change the error rate and thus in principle, if a user has low-depth NGS data they could call genotypes and apply Albrechtsen *et al.* (2009) with an increased error rate to account for the high uncertainty in the genotypes caused by the low-depth nature of the NGS data. Following the settings used to create our proxy truth in section 1.2.1, we estimated α with `calc.a=FALSE` and for all pairs except for the full sibling pair we set $k_2 = 0$ with `fix.k2=0`. Otherwise default parameter settings were used and the Viterbi algorithm was used to infer IBD states.

Assessing inference output
We compared the IBD state inferred from the genotypes called from low-depth NGS data to the results of applying Albrechtsen *et al.* (2009) to the HQ genotype data. Specifically, we evaluated the accuracy, false positive and false negative rates, calculating these values in the same manner as before (section 1.2.2).

Results
The parameter estimates of $R = (k_0, k_1, k_2)$ for each sequencing depth and error rate are displayed in Fig. S4, and the IBD state inference performance is shown in Fig. S5. We observe that increased error rates

tend to improve performance, however the optimal error rate varies across both related pair and sequencing depth.

1.2.5 Application of Albrechtsen *et al.* (2009) to genotypes called from low-depth NGS data (using GATK)

Input Data
To investigate whether Albrechtsen *et al.* (2009) might perform better if applied to genotypes called from low depth NGS data with GATKs multi-sample caller instead of ANGSD we also performed joint genotype calling on the 101 low depth LWK individuals with GATK, and then inferred IBD state with Albrechtsen *et al.* (2009). We tested sequencing depths of 1X, 2X, 4X, and full coverage.

We performed genotype calling on the LWK individuals with GATK in five steps, following best practices.

Step 1: Preliminary per-sample genotypes are called with GATK HaplotypeCaller with output mode `-ERC GVCF` to output per-sample gvcf files.

Step 2: Per-sample gvcf files are combined using GATK GenomicsDBImport.

Step 3: Joint sample genotype calling is performed using GATK GenotypeGVCFs, with the option `--max-alternate-alleles 3`. This option was used to reduce runtime and was recommended by the GATK support team.

Step 4: Variant quality scores are recalibrated with GATK VariantRecalibrator. Following GATK best practicies, we used trache values of `100.0, 99.95, 99.9, 99.8, 99.6, 99.5, 99.4, 99.3, 99.0, 98.0, 97.0, 90.0`, annotations `QD, MQRankSum, ReadPosRankSum, FS, MQ, SOR, DP`, and the hapmap, 1000G, and dbsnp resources provided in the GATK resource bundle.

Step 5: Recalibrated variants are filtered by score with GATK ApplyVQSR, using parameter `--truth-sensitivity-filter-level 99.0`. This produces the final set of called genotypes.

Inference using Albrechtsen *et al.* (2009)
SNPs called with GATK were filtered to the 125,058 loci used in the HQ genotype dataset. Using these genotypes, at each of the four sequencing depths we applied Albrechtsen *et al.* (2009) to the five related pairs. We also tested 6 different error rates, $\epsilon = 0.01, 0.02, 0.05, 0.1, 0.2, 0.5$. We followed the settings used to create our proxy truth in section 1.2.1: we estimated α with `calc.a=FALSE`, and for all pairs except for the full sibling pair we set $k_2 = 0$ with `fix.k2=0`. Otherwise default parameter settings were used and the Viterbi algorithm was used to infer IBD states.

Assessing inference output
We compared the inferred IBD state to the HQ genotype data. Specifically, we evaluated the accuracy, false positive and false negative rates, calculating these values in the same manner as before (section 1.2.2).

Results
The parameter estimates of $R = (k_0, k_1, k_2)$ for each sequencing depth and error rate are displayed in Fig. S2, and the IBD state inference performance is shown in Fig. S3.

1.2.6 Application of hap-IBD to genotypes called from low-depth NGS data

Input data
To investigate the performance of hap-IBD compared to LocalNgsRelate when only low depth NGS data is available we applied hap-IBD genotypes called from NGS data of each of the four sequencing depths at the 125,058 SNPs in the HQ genotype data. As hap-IBD requires phased genotypes, we produced the input data using GLIMPSE [4], which imputes and phases genotypes from genotype likelihoods in low-depth NGS data. We did this in two steps: 1) estimating genotype likelihoods and 2) imputing and phasing those genotype likelihoods with GLIMPSE.

Step 1: GLIMPSE relies on a reference panel, so here we used 1000 Genomes with individuals from LWK removed. We filtered this reference panel to include bi-allic SNP loci with a minor allele frequency greater than 10% based on personal communication with Simone Rubinacci, University of Lausanne. Next, at the remaining loci in the reference panel, we estimated genotype likelihoods for all 101 LWK individuals at full sequencing depth, as well as the downsampled depths of 4X, 2X, and 1X. For this we used ANGSD with the flags `-doBcf 1`, `-doGeno -4`, `-doMajorMinor 3`, `-GL 1`, `-doMaf 1`, `-doCounts 1`, `-MinMapQ 25`, and `-minQ 20`.

Step 2: GLIMPSE, which phases and imputes genotype likelihoods, is run in four steps. The first step uses the tool “chunk” and divides the genome into blocks. We used the same settings as the method’s preprint, `--window-size 2000000`, `--window-count 2000`, `--buffer-size 200000`, and `--buffer-count 200`. Next, each chunk was imputed and phased with the tool “phase”, using the reference panel described above. Here we set the number of iterations to be the maximum allowed with `--main 15`. Otherwise default parameters were used. The phased chunks were then merged using the tool “ligate” and default parameters. Finally, phased and imputed genotypes were generated with the tool “sample” and default parameters.

Inference using hap-IBD
The phased and imputed genotypes were subsequently used to call IBD segments with hap-IBD, using `min-extend=0.2` as recommended for sequencing data, and otherwise default parameters.

Assessing inference output
hap-IBD outputs detected IBD segments. To compare the results from hap-IBD to the IBD state assigned by Albrechtsen *et al.* (2009) in the HQ genotype data, we took each SNP in the HQ genotype data and determined the number of overlapping IBD segments, as reported from hap-IBD, which we translated into IBD state 0, 1 or 2. Then we compared the IBD state to the results of applying Albrechtsen *et al.* (2009) to the HQ genotype data and calculated accuracy, false positive rate, and false negative rate based on this comparison, as in previous sections (section 1.2.2).

Results
The method’s performance is reported in the main text’s Fig. 1. Surprisingly, we observe that as sequencing depth increases, accuracy decreases and the false negative rate increases. Personal communication with the authors of GLIMPSE discussing this effect failed to diminish it.

1.2.7 Using allele frequency estimates from other populations

Input Data
So far we have always estimated allele frequencies from the same population that our related pairs are from (LWK). It is not always the case, however, that users will be able to estimate allele frequencies from the same population as their samples. Here we test the performance of LocalNgsRelate when we estimate allele frequencies from two other populations of differing distance from LWK: YRI, another African population like LWK, and CEU, a European population. For both populations allele frequencies were calculated from the HQ genotype data downloaded from the same server as Section 1.1.2. 189 individuals were used to estimate allele frequencies for YRI, and 183 were used for CEU. As input data for the LWK samples we use the same genotype likelihoods in our related pairs as estimated previously (Section 1.2.2).

Calculating allele frequencies
To reduce error due to low depth sequencing data, we estimate allele frequencies from HQ genotype data for individuals from YRI and CEU. For each population, we use the command `--freq` from `plink 1.9` to estimate allele frequencies at the same 125,058 loci which the genotype likelihoods were calculated at.

Inference using LocalNgsRelate
IBD state was infered and evaluated using the same proceedure as in Section 1.2.2.

Results

The performance of LocalNgsRelate for each related pair, over a range of sequencing depths, and using allele frequencies from either YRI or CEU is shown in figure S7.

2 Supplementary Tables

AA	Aa	aa
$(f_l^A)^2$	$2f_l^A f_l^a$	$(f_l^a)^2$

Table S1: $P(G_l^i|f_l^A)$. The probability that the true genotype of individual i at locus l is G_l^i given that the population frequency of allele A at locus l is $f_l^A = 1 - f_l^a$.

G_l^i	G_l^j	X_l=0	X_l=1	X_l=2
AA	AA	$(f_l^A)^2$	f_l^A	1
AA	Aa	$2f_l^A f_l^a$	f_l^a	0
AA	aa	$(f_l^a)^2$	0	0
Aa	AA	$(f_l^A)^2$	$\frac{1}{2}f_l^A$	0
Aa	Aa	$2f_l^A f_l^a$	$\frac{1}{2}f_l^A + \frac{1}{2}f_l^a$	1
Aa	aa	$(f_l^a)^2$	$\frac{1}{2}f_l^a$	0
aa	AA	$(f_l^A)^2$	0	0
aa	Aa	$2f_l^A f_l^a$	f_l^A	0
aa	aa	$(f_l^a)^2$	f_l^a	1

Table S2: $P(G_l^j|f_l^A, X_l = m, G_l^i)$ for $m \in \{0, 1, 2\}$. The probability that the true genotype of individual j at locus l is G_l^j, given that at locus l the true genotype of individual i is G_l^i, that i and j share X_l alleles IBD and that the population frequency of allele A is $f_l^A = 1 - f^a$.

Relationship	Sample 1	Sample 2	Depth 1	Depth 2
Parent offspring (PO)	NA19313	NA19331	3.6	7.1
Full sibling (FS)	NA19331	NA19334	7.1	6.0
Half sibling (HS)	NA19027	NA19042	6.9	7.6
First cousin (C1)	NA19451	NA19452	4.5	4.5
Unrelated (UR)	NA19313	NA19027	3.6	6.9

Table S3: The five pairs of individuals used to test LocalNgsRelate.

	PO		FS		HS		C1		UR	
	k_0, k_1	α	k_0, k_1, k_2	α	k_0, k_1	α	k_0, k_1	α	k_0, k_1	α
Calculate α	0, 1	0.0131	0.28, 0.56, 0.15	0.0616	0.64, 0.36	0.0326	0.75, 0.25	0.0395	0.99, 0.01	0.1
Estimate α	0, 1	0.001	0.25, 0.47, 0.29	0.144	0.48, 0.52	0.082	0.74, 0.26	0.0723	1, 0	0.001
Limit α	0, 1	0.001	0.24, 0.48, 0.28	0.08	0.48, 0.52	0.08	0.74, 0.26	0.0723	0.98, 0.02	0.08
Double ϵ	0, 1	0.001	0.24, 0.48, 0.28	0.08	0.47, 0.53	0.08	0.74, 0.26	0.0714	0.98, 0.02	0.08

Table S4: Here we evaluate the effect of different parameter settings in Albrechtsen *et al.* (2009) on the output. We run Albrechtsen *et al.* (2009) on the cleaned and pruned array data for each of the five pairs and report the estimates of parameters $R = (k_0, k_1, k_2)$ (for details see Section 1.2.1).

3 Supplementary Figures

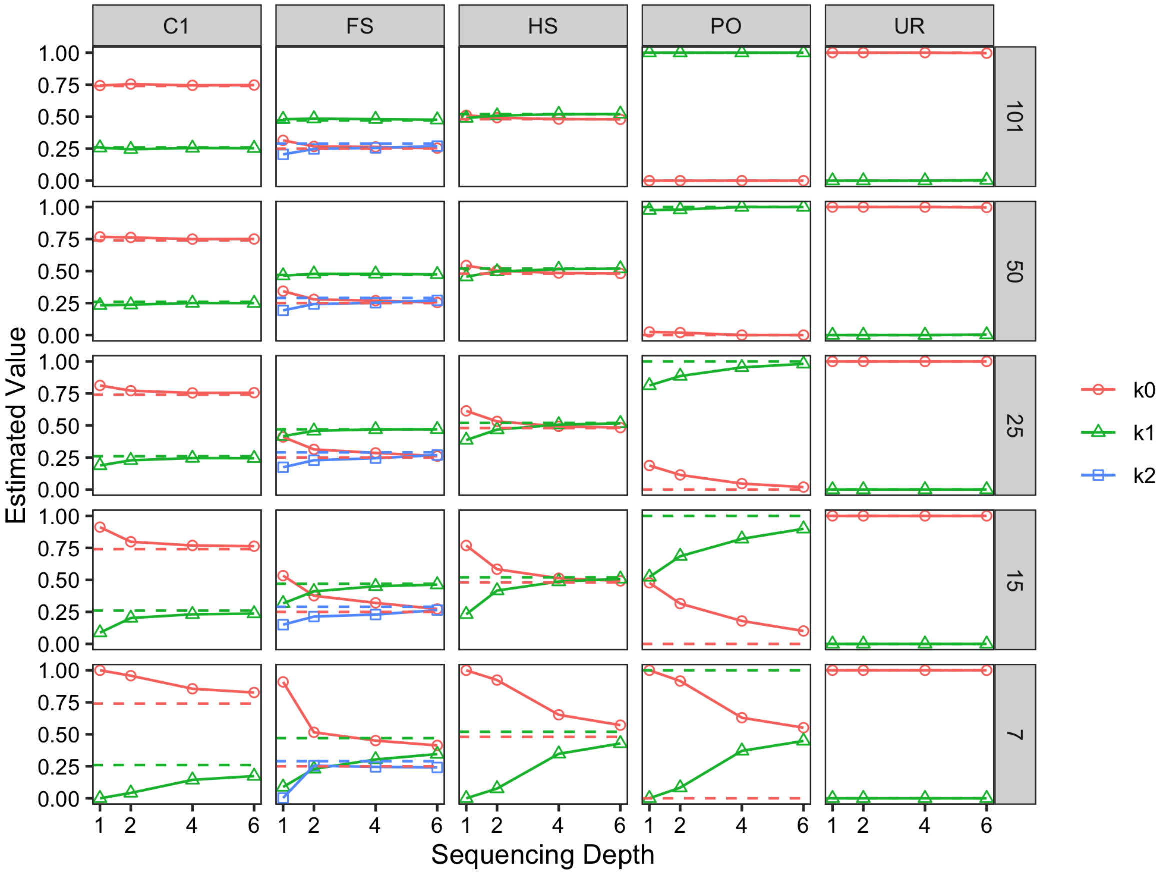

Figure S1: Assessment of LocalNgsRelate's ability to estimate $R = (k_0, k_1, k_2)$ for five pairs of individuals using different sample sizes for allele frequency estimation and different sequencing depths. The results were obtained by downsampling low-depth whole genomes from ∼6X to 4X, 2X, and 1X, and for each of these sequencing depths we then estimated genotype likelihoods at SNP loci which have been pruned for LD (for details see Section 1.2.2). Finally, we used LocalNgsRelate to estimate $R = (k_0, k_1, k_2)$ for all the different sequencing depths and using five different sample sizes for allele frequency estimation. The dashed lines represent the values of k_0, k_1 and k_2 estimated by Albrechtsen *et al.* (2009) from the HQ genotype data (our proxy for the true R).

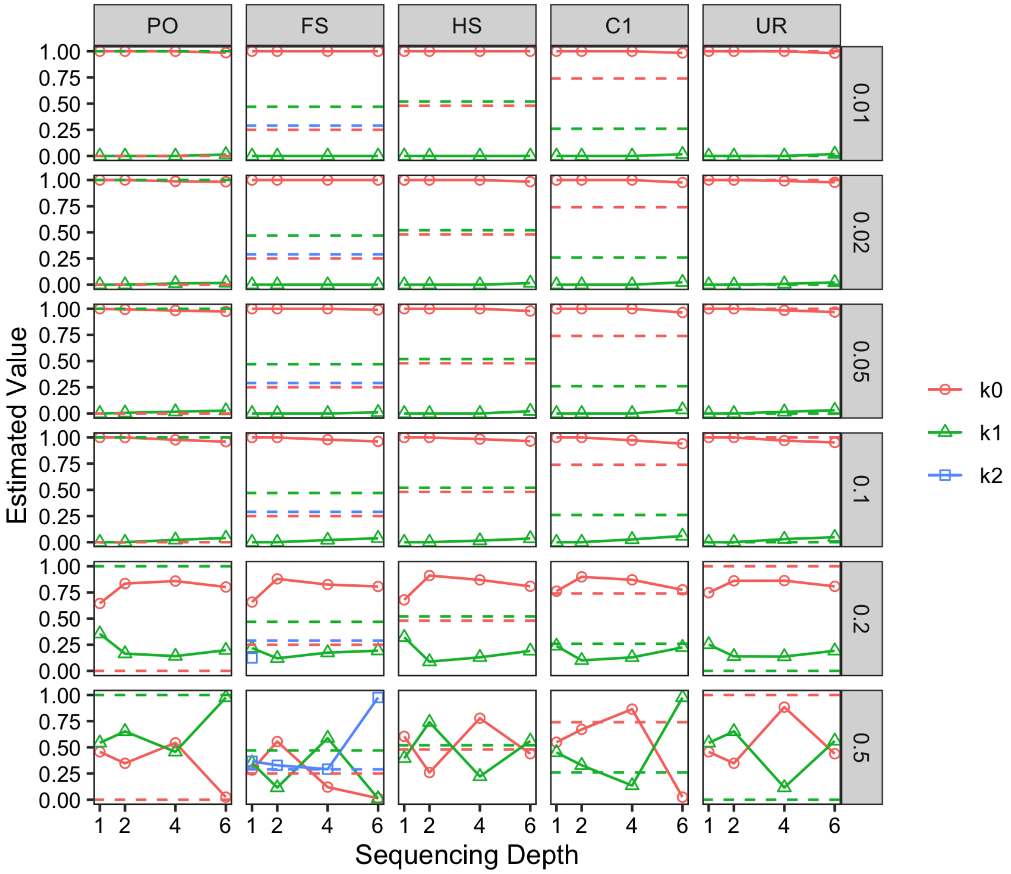

Figure S2: Estimates of $R = k0, k1, k2$ over a range of error rates by Albrechtsen *et al.* (2009) from genotypes called with GATK. Dashed lines represent estimates of R in HQ genotype data. For details on genotype calling using GATK or IBD inference with Albrechtsen *et al.* (2009), see section 1.2.5.

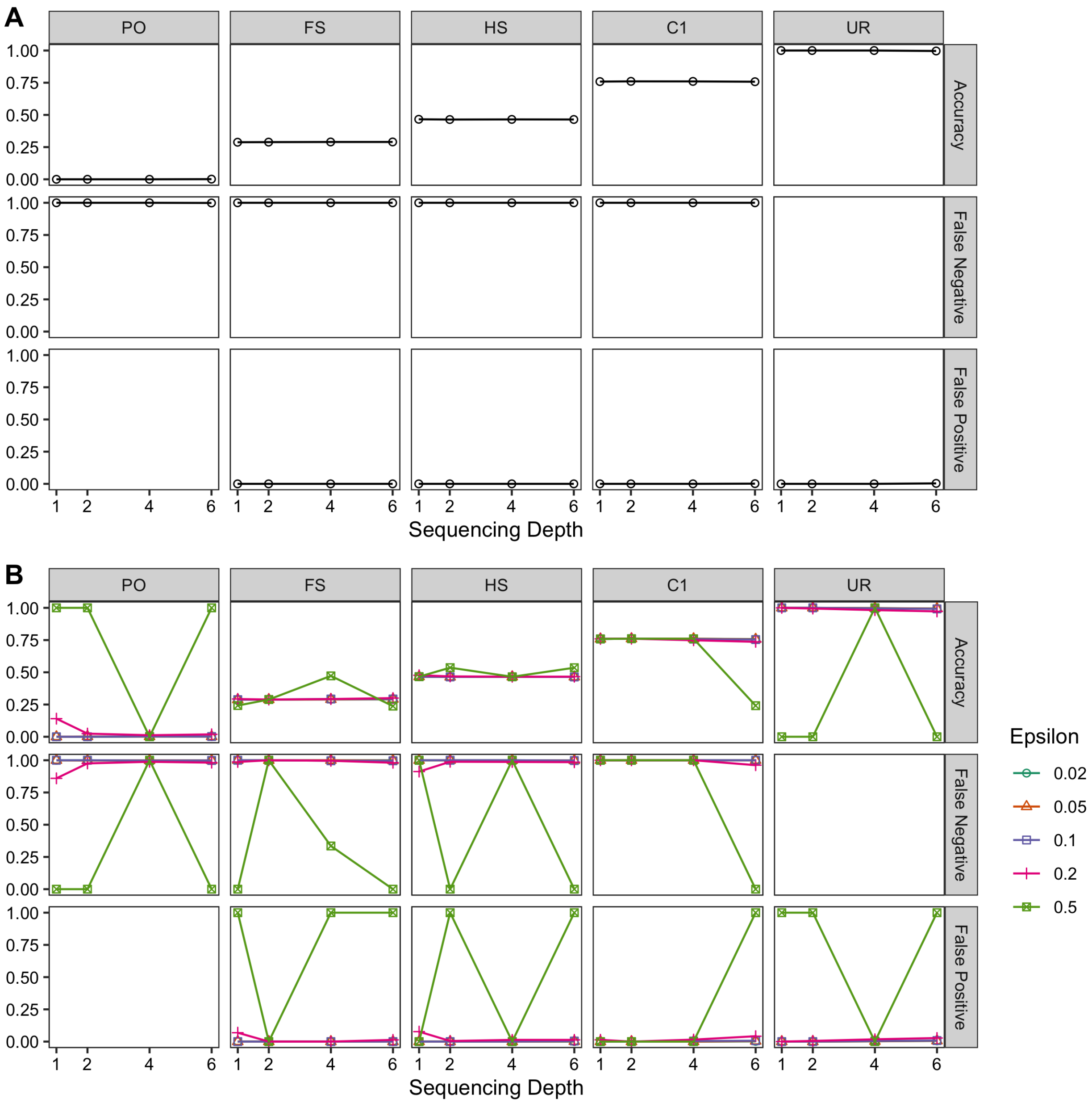

Figure S3: Performance of Albrechtsen *et al.* (2009) applied to genotypes called from low-depth NGS data using GATK. In the main Figure 1 the equivalent results were obtained by applying the same method to genotypes called from low-depth NGS data using ANGSD rather than GATK. Here we explore if using the multisample caller in GATK could improve the performance of Albrechtsen *et al.* (2009). This was not the case. **A.** Results when using the default value of $\epsilon = .01$ when running Albrechtsen *et al.* (2009). **B.** Results when testing a range of epsilon values.

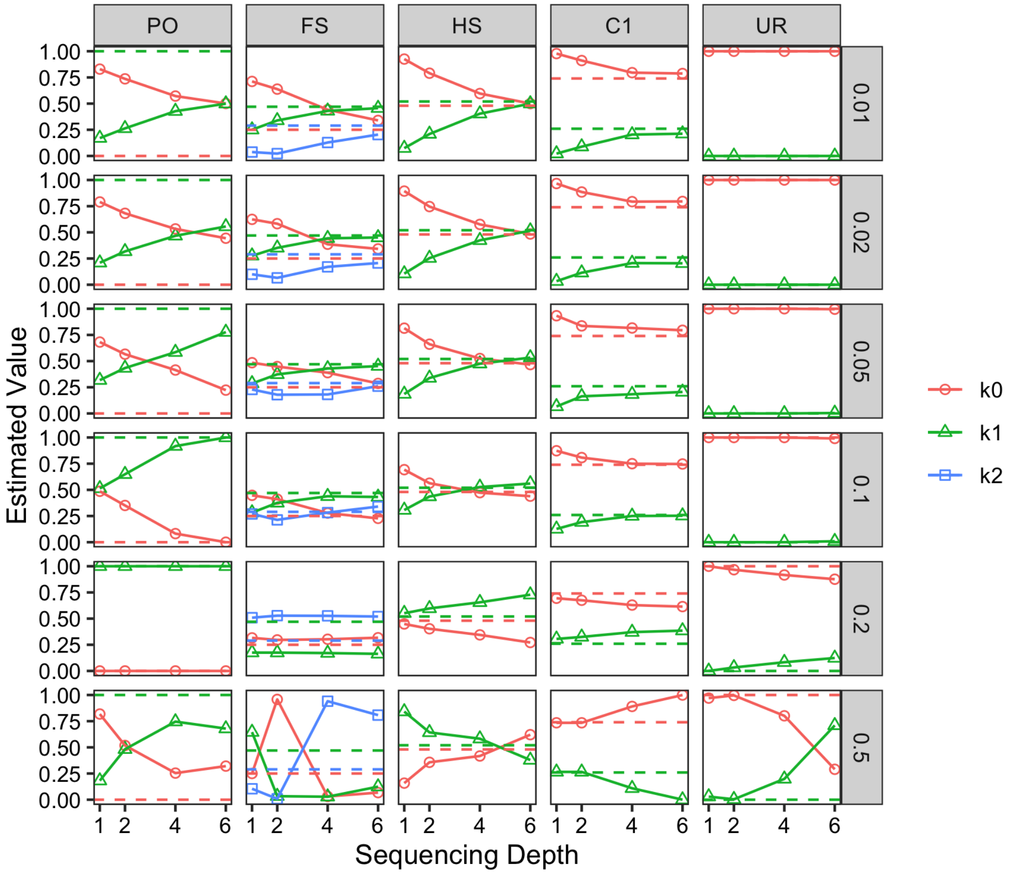

Figure S4: Assessment of Albrechtsen *et al.* (2009)'s estimation of $R = (k_0, k_1, k_2)$, using genotypes called with ANGSD from low-depth NGS data and different genotype error rates ϵ. Here we took the low-depth NGS data and downsampled from ~6X to 4X, 2X, and 1X. At each sequencing depth we then called genotypes at SNP loci, which had been pruned for LD (for details see Section 1.2.4). We used Albrechtsen *et al.* (2009) to estimate $R = (k_0, k_1, k_2)$ from the called genotypes with different error rates. The dashed lines represent the values of k_0, k_1 and k_2 estimated by Albrechtsen *et al.* (2009) from the HQ genotype data (our proxy for the true R).

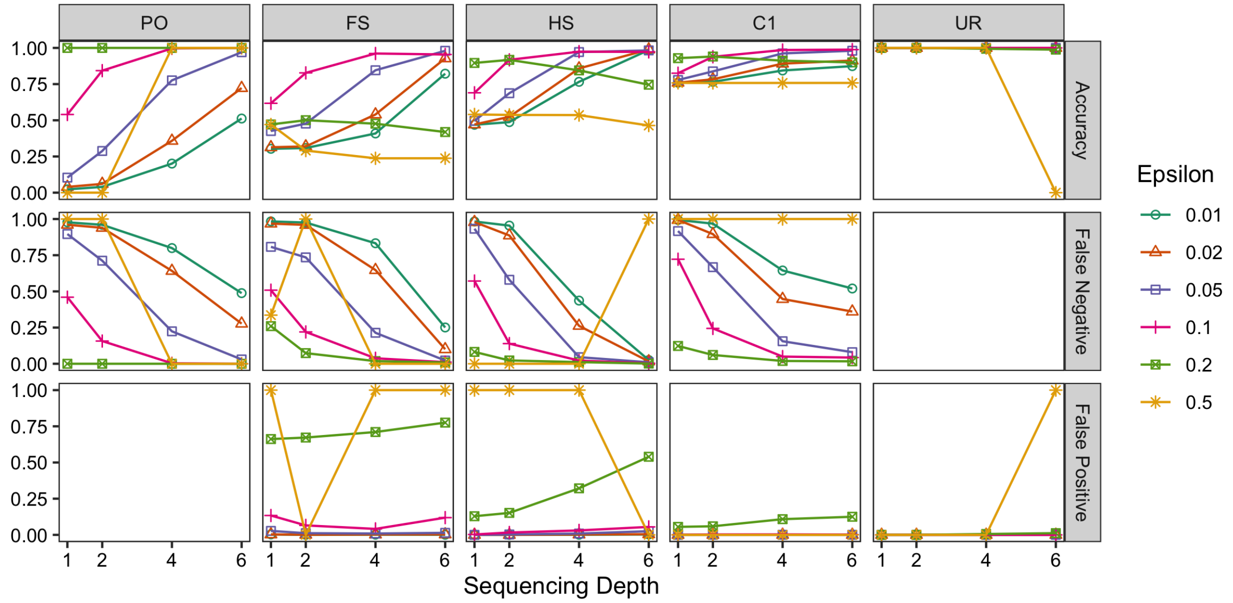

Figure S5: Performance of Albrechtsen *et al.* (2009)'s assignment of IBD state, using genotypes called with ANGSD from low-depth NGS data and different genotype error rates ϵ. Here we took the low-depth genomes and downsampled from ~6X to 4X, 2X, and 1X. For each sequencing depth we then called genotypes at SNP loci, which had been pruned for LD (for details see Section 1.2.4). Finally, we applied Albrechtsen *et al.* (2009) to assign an IBD state at each locus using the Viterbi algorithm to determine the IBD states and compared these to the IBD states inferred by applying Albrechtsen *et al.* (2009) to the HQ genotype data (our proxy for the true states).

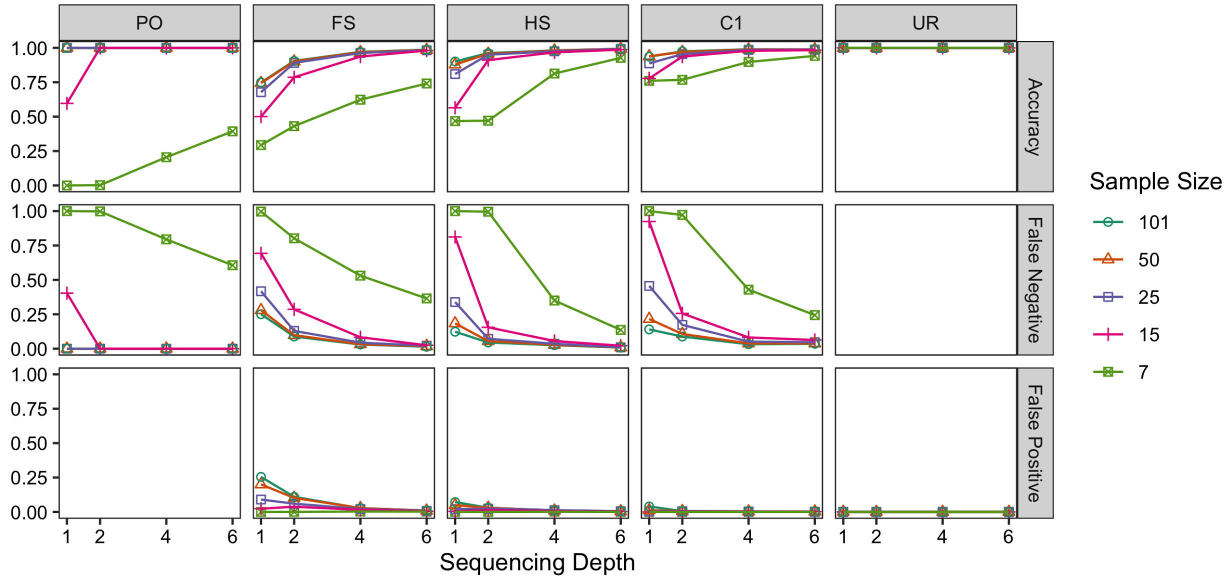

Figure S6: Performance of LocalNgsRelate's assignment of IBD state for five pairs of individuals over a range of sample sizes for allele frequency estimation and sequencing depths. The results were obtained by downsampling low-depth whole genomes from ∼6X to 4X, 2X, and 1X, and for each of these sequencing depths we then estimated genotype likelihoods at SNP loci which have been pruned for LD (for details see Section 1.2.2). Finally, we used LocalNgsRelate and the Viterbi algorithm to assign IBD state, and compared this assignment to the IBD state inferred by applying Albrechtsen *et al.* (2009) to the HQ genotype data (our proxy for the true states).

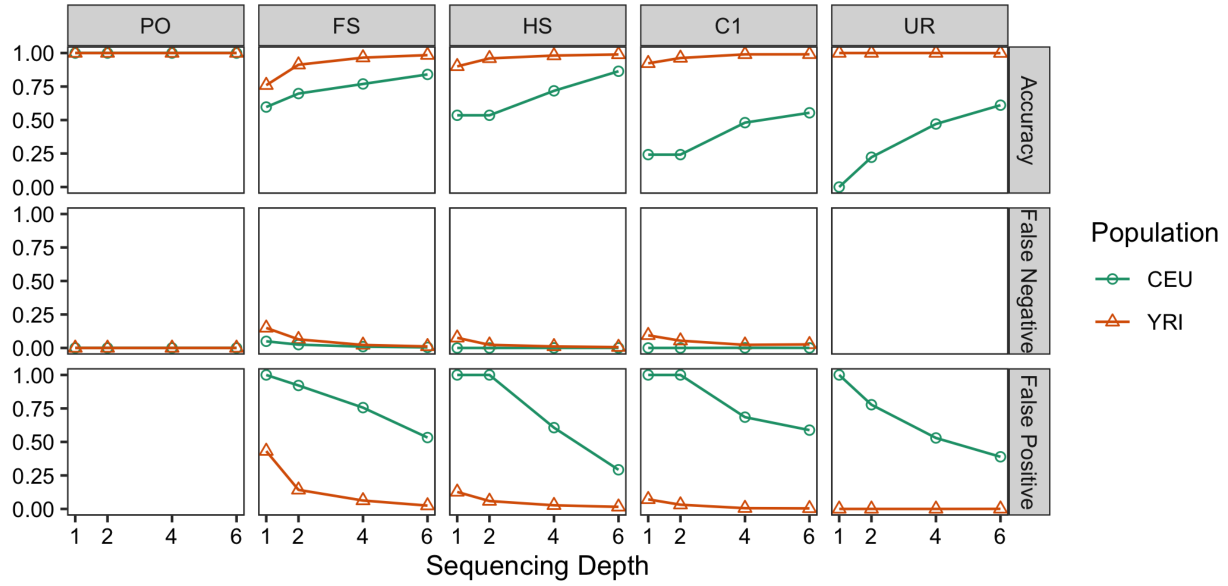

Figure S7: Performance of IBD inference by LocalNgsRelate, using allele frequencies estimated from two alternative populations, YRI and CEU. For methodological details, see section 1.2.7.

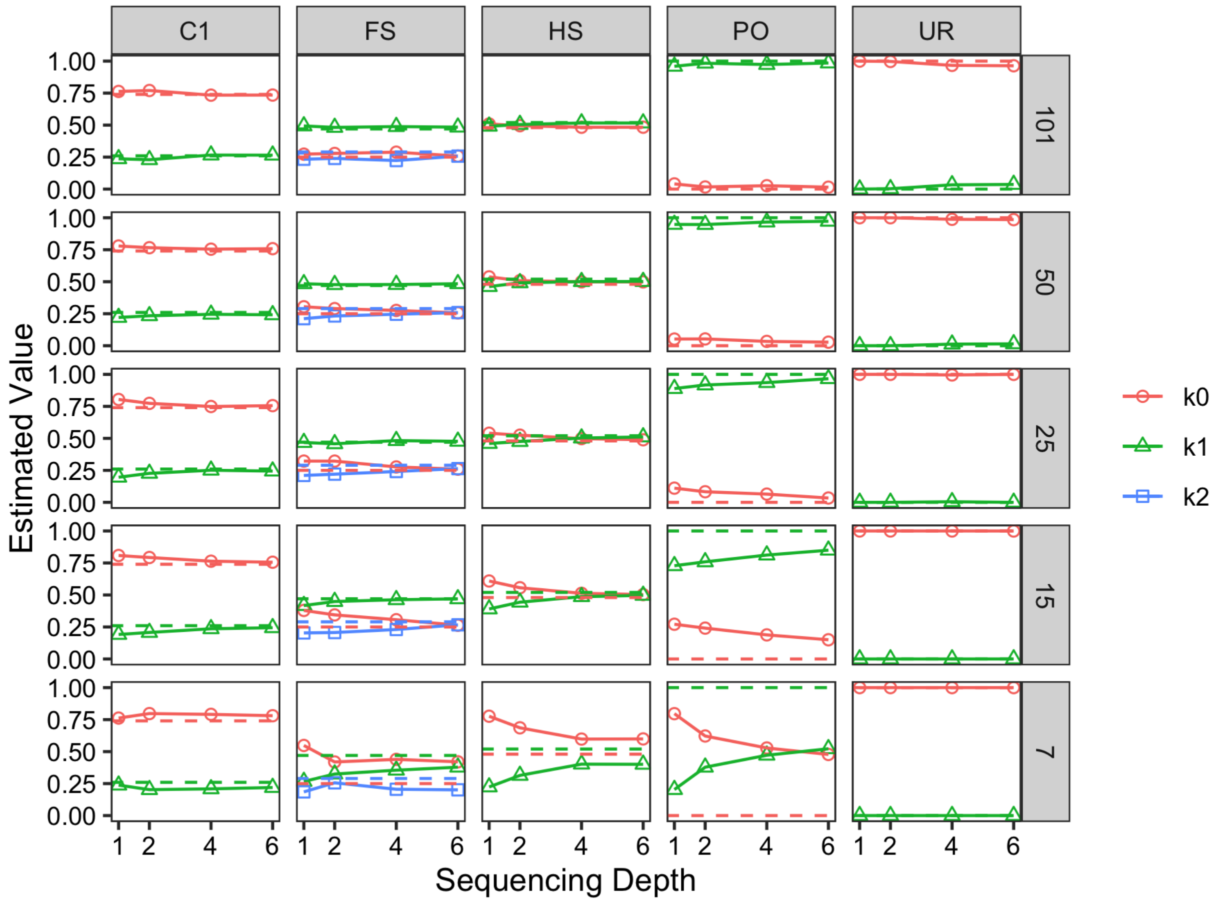

Figure S8: Assessment of LocalNgsRelate's estimation of $R = (k_0, k_1, k_2)$ from GLs for SNPs thinned to be no closer than 20 KB, for the five related pairs over a range of sample sizes and sequencing depths. To prepare the data, we downsampled the low-depth whole genomes from ∼6X to 4X, 2X, and 1X. For each sample size and sequencing depth we then estimated genotype likelihoods and allele frequencies with ANGSD [1]. To approximate pruning for LD, we used plink [3] to thin SNPs to be no closer than 20 KB from each other, resulting in about 125,000 SNPs (for details see Section 1.2.3). Finally, we used LocalNgsRelate to estimate $R = (k_0, k_1, k_2)$ for each sample size at each different sequencing depth. The dashed lines represent the values of $R = (k_0, k_1, k_2)$ estimated by Albrechtsen *et al.* (2009) from the genotype data (our proxy for the true R).

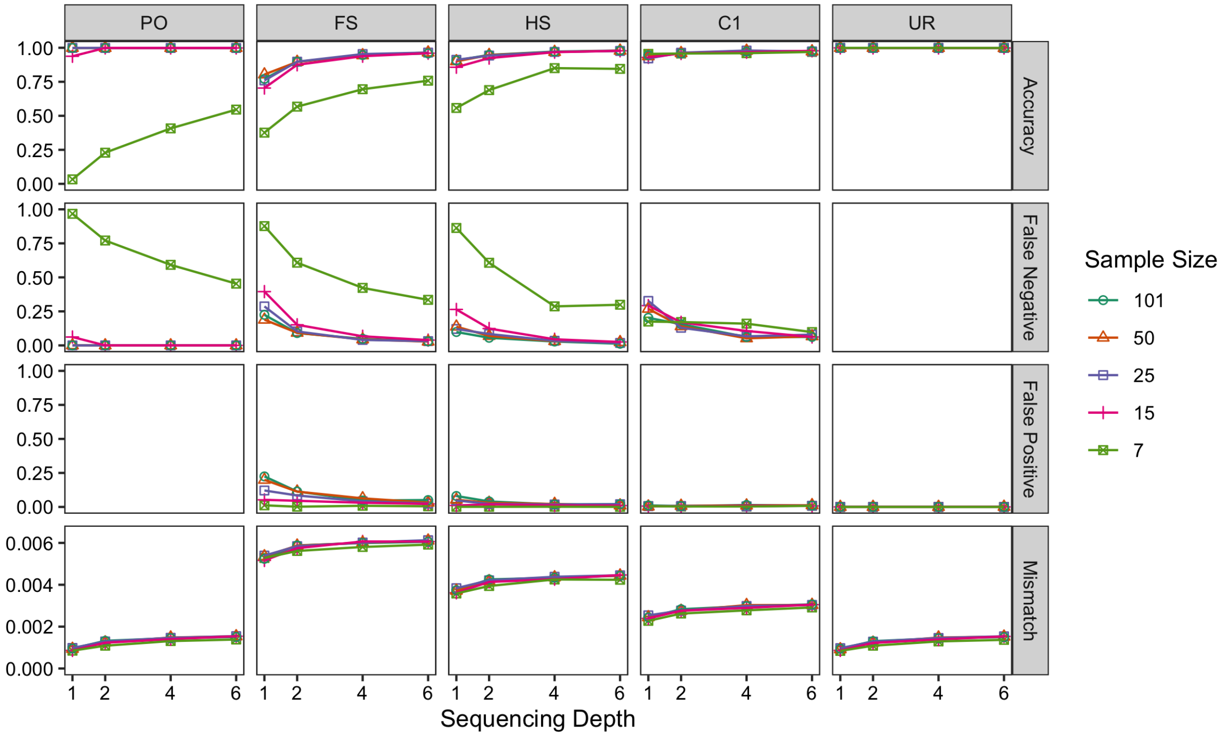

Figure S9: LocalNgsRelate's performance assigning IBD state with the Viterbi algorithm, using GLs for SNPs thinned to be no closer than 20 KB, for the five related pairs over a range of sample sizes and sequencing depths. To prepare the data we first downsampled the low-depth whole genomes from ∼6X to 4X, 2X, and 1X. For each sample size and sequencing depth we then estimated genotype likelihoods and allele frequencies with ANGSD [1]. To approximate pruning for LD, we used plink [3] to thin SNPs to be no closer than 20 KB from each other, resulting in about 125,000 SNPs (for details see Section 1.2.3). Finally, we used LocalNgsRelate to call an IBD state at each SNP locus using the Viterbi algorithm and compared those calls to the IBD states inferred by applying Albrechtsen *et al.* (2009) to the HQ genotype data (our proxy for the true states).

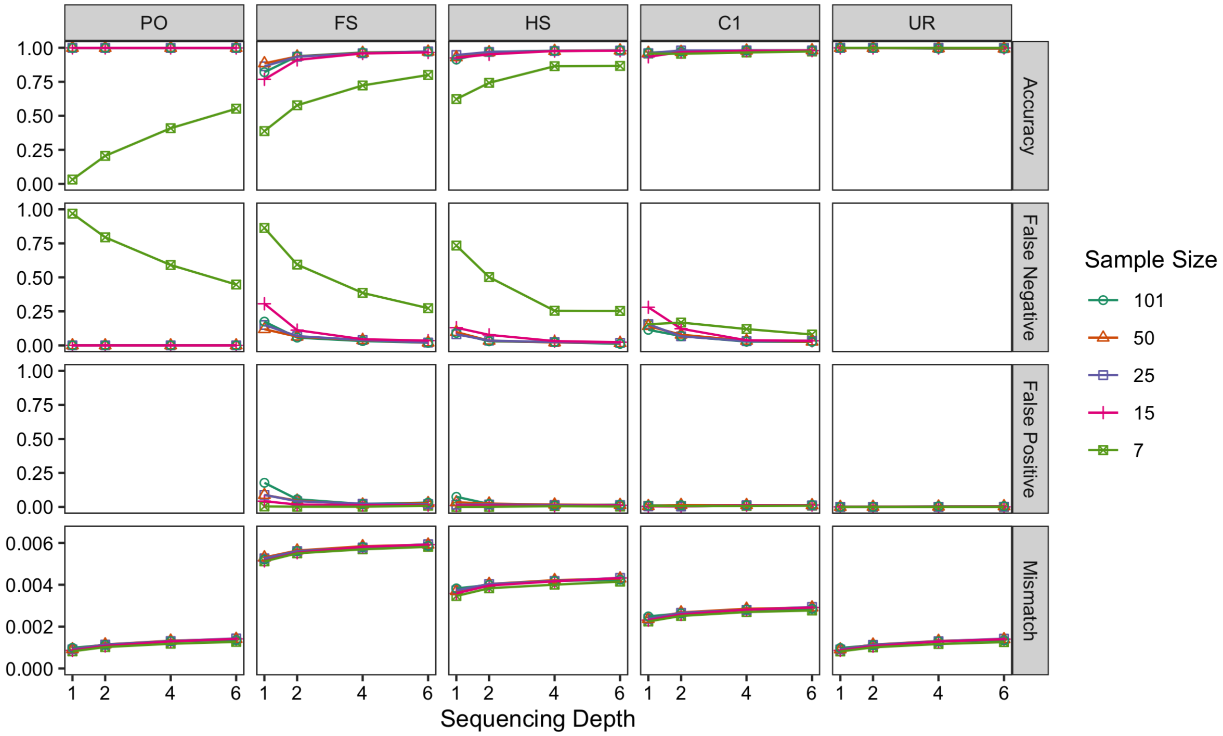

Figure S10: LocalNgsRelate's performance assigning IBD state with the Viterbi algorithm, using GLs for SNPs thinned to be no closer than 10 KB, for the five related pairs over a range of sample sizes and sequencing depths. To prepare the data we first downsampled the low-depth whole genomes from ∼6X to 4X, 2X, and 1X. For each sample size and sequencing depth we then estimated genotype likelihoods and allele frequencies with ANGSD [1]. To approximate pruning for LD, we used plink [3] to thin SNPs to be no closer than 10 KB from each other, resulting in about 250,000 SNPs (for details see Section 1.2.3). Finally, we used LocalNgsRelate to call an IBD state at each SNP locus using the Viterbi algorithm and compared those calls to the IBD states inferred by applying Albrechtsen *et al.* (2009) to the HQ genotype data (our proxy for the true states).

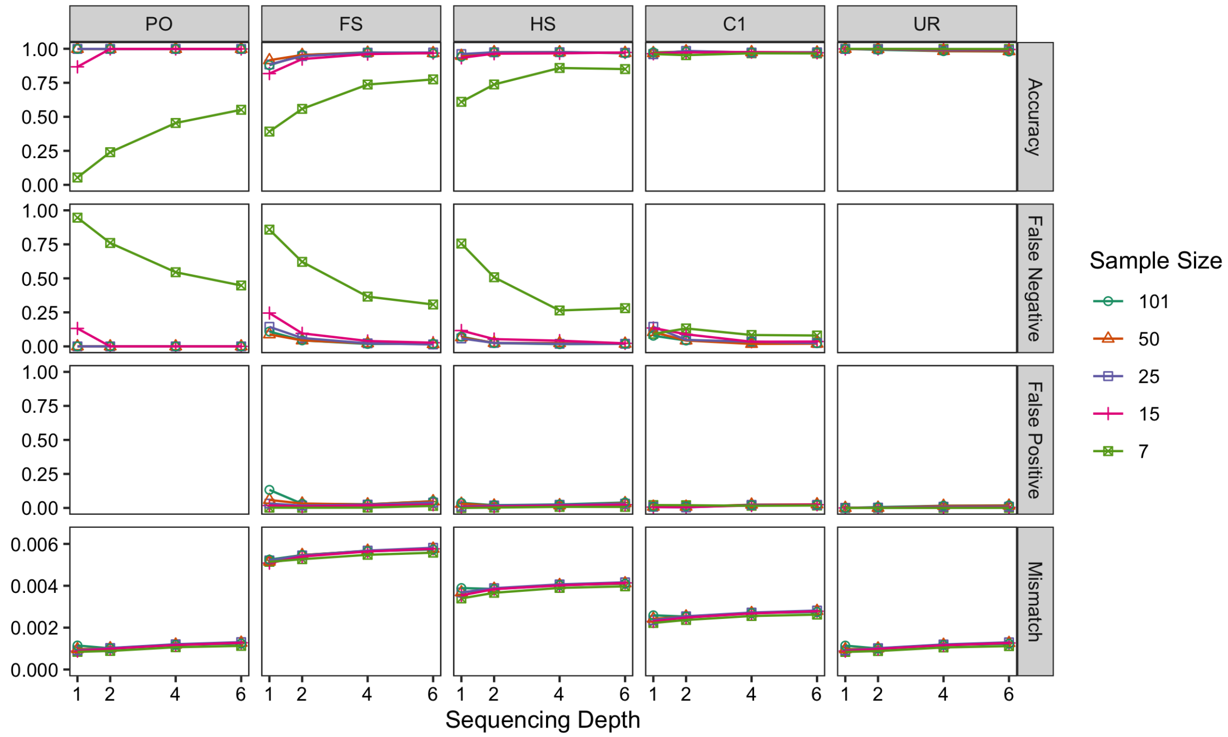

Figure S11: LocalNgsRelate's performance assigning IBD state with the Viterbi algorithm, using GLs for SNPs thinned to be no closer than 5 KB, for the five related pairs over a range of sample sizes and sequencing depths. To prepare the data we first downsampled the low-depth whole genomes from ∼6X to 4X, 2X, and 1X. For each sample size and sequencing depth we then estimated genotype likelihoods and allele frequencies with ANGSD [1]. To approximate pruning for LD, we used plink [3] to thin SNPs to be no closer than 5 KB from each other, resulting in about 500,000 SNPs (for details see Section 1.2.3). Finally, we used LocalNgsRelate to call an IBD state at each SNP locus using the Viterbi algorithm and compared those calls to the IBD states inferred by applying Albrechtsen *et al.* (2009) to the HQ genotype data (our proxy for the true states).

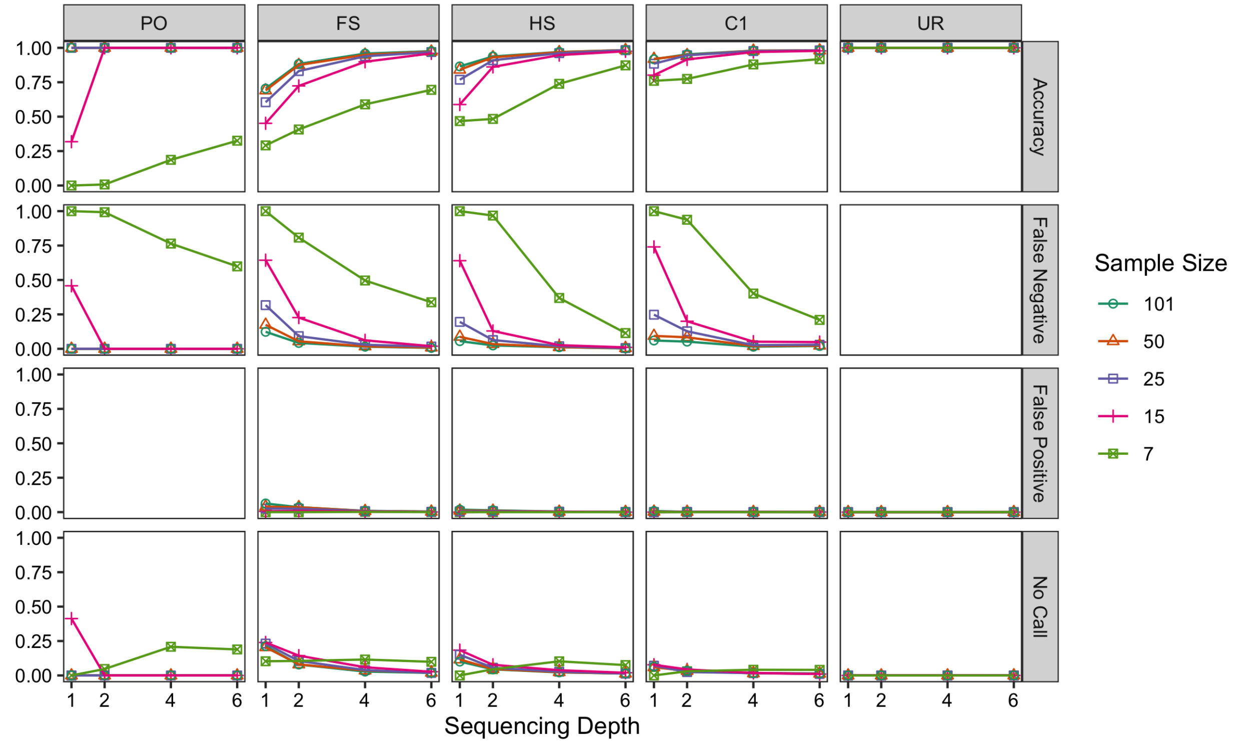

Figure S12: Performance of LocalNgsRelate's assignment of IBD state using posterior decoding and a posterior cutoff of 0.75 for five related pairs over a range of sample sizes and sequencing depths based on GLs for SNPs thinned to be no closer than 20 KB. Here we downsampled low-depth whole genomes from ∼6X to 4X, 2X, and 1X. For each sequencing depth, we then called genotype likelihoods at SNP loci, which had been pruned for LD (for details see Section 1.2.2), and for each sequencing depth and sample size, we estimated allele frequencies. Finally, we used LocalNgsRelate to call IBD state at each SNP locus using posterior decoding combined with a posterior cutoff of 0.75 (for details see Section 1.2.2) and compared the called IBD state to the IBD states inferred by applying Albrechtsen *et al.* (2009) to the HQ genotype data (our proxy for the true states).

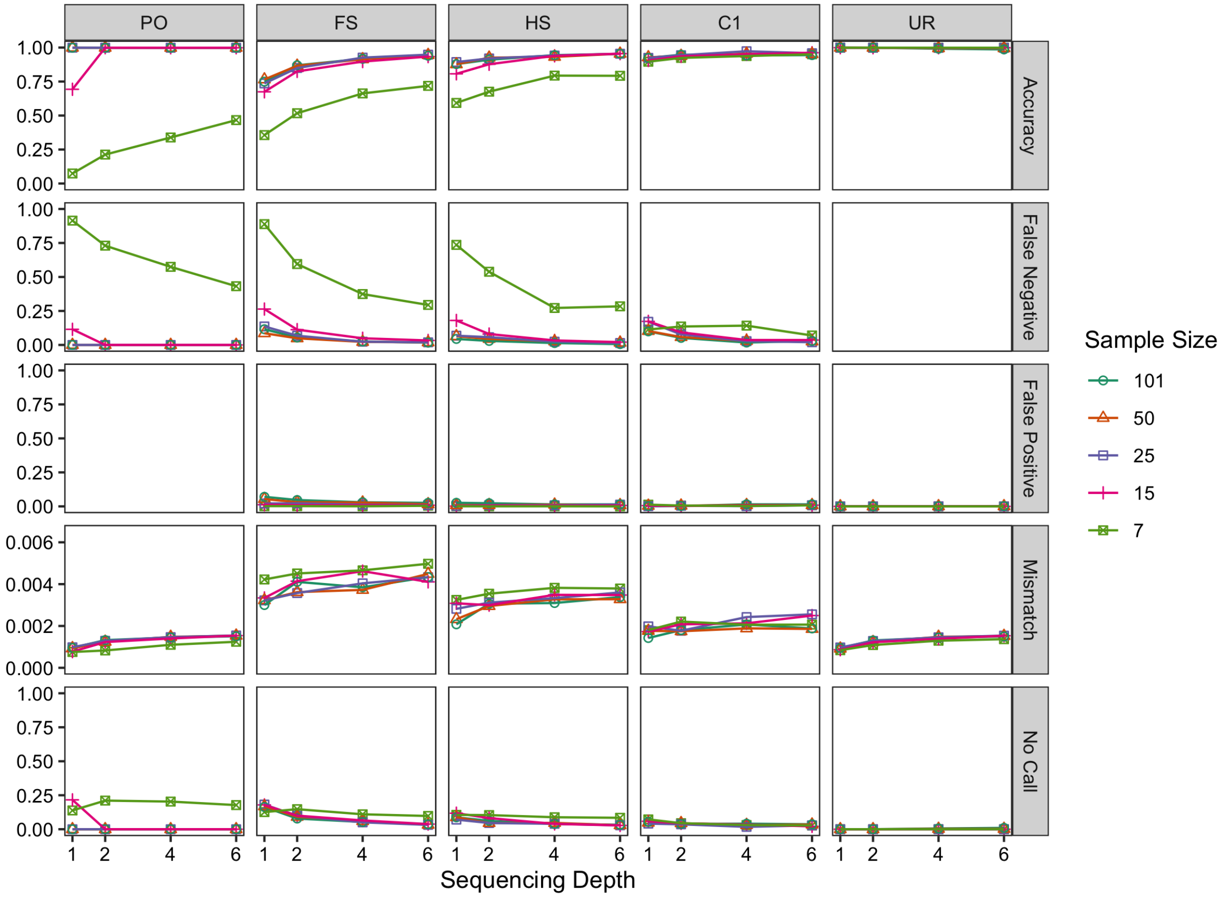

Figure S13: LocalNgsRelate's performance assigning IBD state using posterior decoding and a posterior cutoff of 0.75 for five related pairs over a range of sample sizes and sequencing depths. To prepare the data we first downsampled the low-depth whole genomes from ∼6X to 4X, 2X, and 1X. For each sample size and sequencing depth we then estimated genotype likelihoods and allele frequencies with ANGSD [1]. To approximate pruning for LD, we used plink [3] to thin SNPs to be no closer than 20 KB from each other, resulting in about 125,000 SNPs (for details see Section 1.2.3). Finally, we used LocalNgsRelate to call IBD state at each SNP loci using a posterior cutoff of 0.75, and compare to the IBD state inferred by applying Albrechtsen *et al.* (2009) to the HQ genotype data.

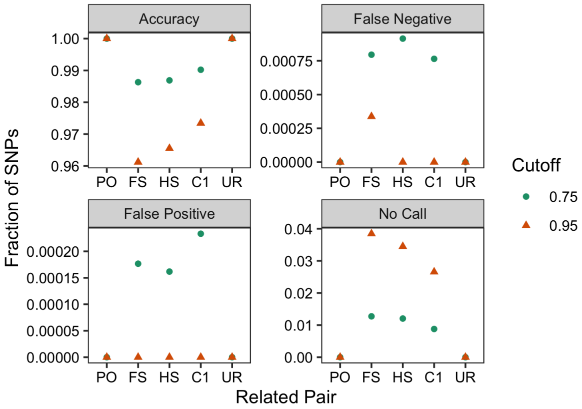

Figure S14: Comparison of results obtained by applying Albrechtsen *et al.* (2009) to LD pruned HQ genotype data using three different IBD state inference methods. For each pair we first performed inference using the Viterbi algorithm and then we compared the results from that to the results obtained using posterior decoding combined with posterior cutoffs of 0.75 and 0.95 (for details see Section 1.2.1).

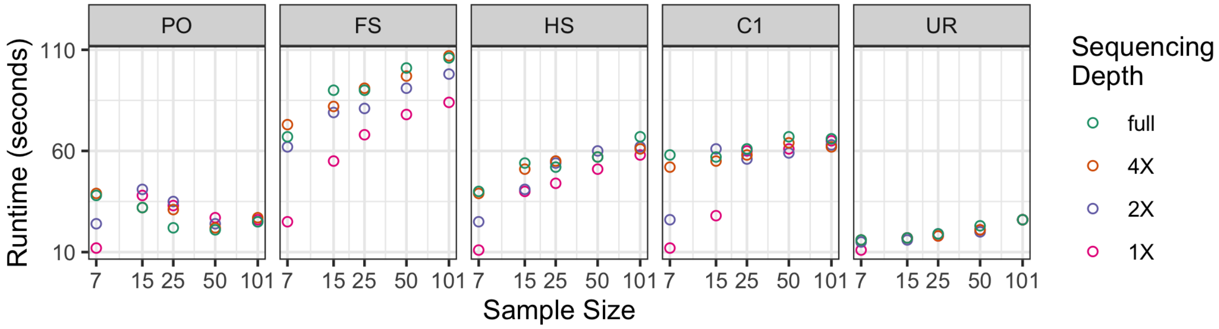

Figure S15: Runtimes for LocalNgsRelate for each related pair, over a range of sample sizes and coverage levels. For each run the method was given 2 Gb of memory.

References

[1] Korneliussen, T. S., Albrechtsen, A., and Nielsen, R. (2014). ANGSD: Analysis of Next Generation Sequencing Data. *BMC Bioinformatics*, **15**, 356.

[2] Li, H., Handsaker, B., Wysoker, A., Fennell, T., Ruan, J., Homer, N., Marth, G., Abecasis, G., Durbin, R., and 1000 Genome Project Data Processing Subgroup (2009). The Sequence Alignment/Map format and SAMtools. *Bioinformatics*, **25**, 2078.

[3] Purcell, S., Neale, B., Todd-Brown, K., Thomas, L., Ferreira, M.A., Bender, D., Maller, J., Sklar, P., De Bakker, P.I., Daly, M.J. and Sham, P.C. (2007). PLINK: a tool set for whole-genome association and population-based linkage analyses. *The American journal of human genetics*, **81**, 559.

[4] Rubinacci, S. and Ribeiro, D.M. and Hofmeister, R. and Delaneau, O. (2020). Efficient phasing and imputation of low-coverage sequencing data using large reference panels. *bioRxiv*.
